# Fasting glucose variability and risk of dementia in Parkinson’s disease: a 9-year longitudinal follow-up study of a nationwide cohort

**DOI:** 10.3389/fnagi.2023.1292524

**Published:** 2024-01-03

**Authors:** Sung Hoon Kang, Yunjin Choi, Su Jin Chung, Seok-Joo Moon, Chi Kyung Kim, Ji Hyun Kim, Kyungmi Oh, Joon Shik Yoon, Sang Won Seo, Geum Joon Cho, Seong-Beom Koh

**Affiliations:** ^1^Department of Neurology, Korea University Guro Hospital, Korea University College of Medicine, Seoul, Republic of Korea; ^2^Biomedical Research Institute, Korea University Guro Hospital, Korea University College of Medicine, Seoul, Republic of Korea; ^3^Department of Neurology, Myongji Hospital, Hanyang University College of Medicine, Goyang, Republic of Korea; ^4^Smart Healthcare Center, Korea University Guro Hospital, Korea University College of Medicine, Seoul, Republic of Korea; ^5^Department of Physical Medicine and Rehabilitation, Korea University Guro Hospital, Seoul, Republic of Korea; ^6^Department of Neurology, Samsung Medical Center, Sungkyunkwan University School of Medicine, Seoul, Republic of Korea; ^7^Alzheimer’s Disease Convergence Research Center, Samsung Medical Center, Seoul, Republic of Korea; ^8^Department of Obstetrics and Gynecology, Korea University Guro Hospital, Korea University College of Medicine, Seoul, Republic of Korea

**Keywords:** Parkinson’ s disease, Parkinson’s disease dementia, glucose variability, fasting glucose, risk factors

## Abstract

**Background:**

Diabetes is associated with an increased risk of Parkinson’s disease dementia (PDD); however, it is unknown whether this association is dependent on continuous hyperglycemia, hypoglycemic events, or glycemic variability. We aimed to investigate the relationship between visit-to-visit fasting glucose variability and PDD development in patients with Parkinson’s disease (PD).

**Methods:**

Using data from the Korean National Health Insurance Service, we examined 9,264 patients aged ≥40 years with *de novo* Parkinson’s disease (PD) who underwent ≥3 health examinations and were followed up until December 2019. Glucose variability was measured using the coefficient of variation, variability independent of the mean, and average real variability. Fine and Gray competing regression analysis was performed to determine the effect of glucose variability on incident PDD.

**Results:**

During the 9.5-year follow-up period, 1,757 of 9,264 (19.0%) patients developed PDD. Patients with a higher visit-to-visit glucose variability had a higher risk of future PDD. In the multivariable adjusted model, patients with PD in the highest quartile (subdistribution hazard ratio [SHR] = 1.50, 95% CI 1.19 to 1.88), quartile 3 (SHR = 1.29, 95% CI 1.02 to 1.62), and quartile 2 (SHR = 1.30, 95% CI 1.04 to 1.63) were independently associated with a higher risk of PDD than those in the lowest quartile.

**Conclusion:**

We highlighted the effect of long-term glucose variability on the development of PDD in patients with PD. Furthermore, our findings suggest that preventive measures for constant glucose control may be necessary to prevent PDD.

## Introduction

Parkinson’s disease (PD) is a common neurodegenerative disease characterized by the deterioration of motor function (Ko et al., 2022). Patients with PD may also exhibit non-motor symptoms, including cognitive dysfunction. The incidence of Parkinson’s disease dementia (PDD) has been increasing because of longer survival resulting from advanced treatment methods (Kang et al., 2023). PDD leads to a reduced quality of life and poor prognosis; therefore, identifying modifiable risk factors for PDD is important for designing strategies to prevent the development of PDD in patients with PD.

Growing evidence has shown that patients with diabetes have a higher incidence of PD and dementia, including Alzheimer’s disease dementia and vascular dementia (Curb et al., 1999; Chornenkyy et al., 2019; Cheong et al., 2020). Several studies have proposed that PD patients with diabetes may have an increased risk of cognitive dysfunction and PDD. Cross-sectional studies have shown diabetes to be independently associated with more severe cognitive dysfunction in PD (Bohnen et al., 2014; Giuntini et al., 2014). In one longitudinal study, PD patients with diabetes had a greater progression of cortical atrophy, leading to cognitive decline (Ong et al., 2017). One meta-analysis found that diabetes increased the risk of PDD (Guo et al., 2019). However, it remains unknown whether this association is dependent on continuous hyperglycemia, hypoglycemic events, or glycemic variability.

Recently, several studies have suggested an association between higher glucose variability and oxidative stress, inflammatory processes, and insulin resistance (Monnier et al., 2006; Ceriello et al., 2008), leading to cardiovascular disease and mortality regardless of baseline glucose levels (Gorst et al., 2015; Wang et al., 2017). Previous studies have also reported that patients with higher glucose variability have an increased risk of incident dementia and PD, irrespective of the presence of diabetes (Lee et al., 2018; Chung et al., 2021). Given these findings, higher glucose variability may be an independent predictor of PDD in patients with PD.

Using the Korean National Health Insurance Service (KNHIS) data, our study aimed to investigate the association between visit-to-visit fasting glucose variability and the risk of incident PDD in patients with PD. We hypothesized that higher glucose variability might be an independent risk factor for PDD due to its shared pathogenesis with vascular dementia and primary degeneration in the subcortical regions (Pillon et al., 1993).

## Methods

### Standard protocol approvals, registrations, and patient consents

This study was approved by the Institutional Review Board of the Korea University Guro Hospital and adhered to the principles of the Declaration of Helsinki. Anonymous and de-identified information was used for analysis; therefore, informed consent was not obtained.

### Date source

We used a customized dataset from the KNHIS, which includes more than 99% of the Korean population (approximately 50 million individuals).^1^ The KNHIS database includes personal information, health insurance claim codes (procedures and prescriptions), diagnostic codes from the Korean Standard Classification of Diseases, 7th Revision, based on the International Classification of Diseases, 10th Revision (ICD-10), death records from the Korean National Statistical Office, and general medical examination data for each participant from 2002 to 2019. Data on body mass index (BMI), smoking status, and alcohol consumption were obtained from the general health examinations in the KNHIS database.

### Study participants

Patients with *de novo* PD aged ≥40 years between January 2002 and December 2010 were enrolled based on the ICD-10 code G20 for PD and PD medication prescriptions. A total of 114,848 eligible candidates were identified. We excluded 1,132 patients with a previous history of dementia diagnosis, 3,379 with a diagnosis of dementia within the first year of follow-up to rule out dementia with Lewy bodies, 30,712 with a diagnosis of Parkinson’s syndrome (ICD-10 codes G21, G22, or G23), 68,589 without at least three fasting glucose levels in health examinations between January 2002 and one year after the date of PD diagnosis, and 1,769 with an interval > 1 year between the date of PD diagnosis and the index health examination, which is the nearest examination from the date of PD diagnosis. As a result, 9,264 patients were included in this study.

### Measurements and definition of diseases

The health examination included blood pressure (BP) and BMI measurements, and blood sampling, including fasting glucose and total cholesterol levels at the nearest examination from the date of PD diagnosis (index health examination). Hypertension was defined according to the ICD-10 codes (I10-15) and a prescription of antihypertensive medication or a systolic/diastolic BP ≥ 140/90 mmHg. Diabetes was defined according to the corresponding ICD-10 codes (E8-14) and a prescription of glucose-lowering medication or fasting glucose ≥126 mg/dL. Hyperlipidemia was defined according to ICD-10 code (E78) and a prescription of lipid-lowering medication or total cholesterol level ≥ 240 mg/dL. Smoking status was categorized into three groups: those who had never smoked, ex-smokers, and current smokers. Heavy alcohol consumption was defined as alcohol consumption ≥ three times per week. Regular exercise was defined as performing physical activity ≥ three times per week.

### Definition of glucose variability

Glucose variability was calculated form three or more fasting glucose levels measured at the patients’ health examination between January 2002 and one year after the PD diagnosis date. Three measures of variability were considered (Kang et al., 2022), variability independent of mean (VIM), coefficient of variation (CV), and average real variability (ARV). VIM was calculated by dividing the standard deviation (SD) by the mean raised to the power β (SD/mean^β^), in which β was the regression coefficient based on the PROC NLIN procedure of the SAS package. CV was calculated as the SD divided by the mean. ARV was the average of the absolute differences between consecutive values and was calculated using the following formula, in which N was the number of glucose measurements:


$$ARV=\frac{1}{n-1}\overset{n-1}{\underset{i=1}{\sum }}\left|x_{i+1}-x_{i}\right|\text{.}$$

### Definition of outcome and follow-up

The outcome of the study was the development of PDD (newly diagnosed dementia in patients with PD), which was defined according to the relevant ICD-10 codes (F00, F02, F03, F05, or G30) and prescriptions of dementia medication, including donepezil, rivastigmine, galantamine, and memantine. To exclude Lewy body dementia, we excluded patients diagnosed with dementia within one year after the diagnosis of PD. The competing risk was death, obtained from death records from the Korean National Statistical Office. Patients without PDD during follow-up were considered to have completed the study on the date of death or at the end of follow-up. All patients were followed up from the date of PD diagnosis (baseline) to the date of PDD diagnosis, date of death, or until December 2019.

### Statistical analyses

Baseline characteristics are presented as the mean ± SD or median (interquartile range [IQR]) and frequency (%). The patients were divided into four groups based on the glucose CV quartiles. PDD incidence was examined according to glucose variability. The association between glucose variability and the development of PDD was investigated using a Fine and Gray competing regression model with glucose variability as a separate predictor and death as a competing risk, controlling for age, sex, the presence of hypertension, diabetes and hyperlipidemia, heavy alcohol consumption, smoking, and regular exercise (Model 1). To account for the possible effects of systolic and diastolic BP, fasting glucose, total cholesterol, and BMI at the index health examination, further analyses were performed after controlling for the covariates in Model 1 plus systolic BP, diastolic BP, fasting glucose, total cholesterol, and BMI at the index health examination (Model 2). Sensitivity analyses were used to exclude patients with diabetes to eliminate the effects of diabetes, including continuous hyperglycemia and hypoglycemic events. Furthermore, analyses using other measures of glucose variability, including VIM and ARV, were performed.

All reported *p*-values were two-sided and statistical significance was set at 0.05. All analyses were performed using SAS (version 9.4; SAS Institute, Inc.).

## Results

### Clinical characteristics of the study participants

Patients with PD were classified into four groups based on the glucose CV. CV was normally distributed, and the CV quartiles were defined based on the cut-off points 0.072, 0.112, and 0.168. Table 1 shows the baseline demographics of the PD cohort, presented as the mean ± SD or n (%). Mean age, systolic BP, fasting glucose and total cholesterol, female ratio, frequency of hypertension, diabetes, dyslipidemia, smoking, heavy alcohol consumption, and regular exercise were different across all groups. Of note, patients in the higher quartiles were older and had higher systolic BP, fasting glucose levels, and prevalence of hypertension, diabetes, and hyperlipidemia. However, no significant difference was found in diastolic BP (*p* = 0.491) or BMI (*p* = 0.673). The median follow-up duration was 9.5 (5.7–10.9) years.

**Table 1:** Baseline characteristics of study participants according to CV quartile.

	Quartile 1 (*n* = 2,314)	Quartile 2 (*n* = 2,327)	Quartile 3 (*n* = 2,301)	Quartile 4 (*n* = 2,322)	*p* value
Variables					
Age, years	67.1 ± 9.2	67.3 ± 9.4	67.9 ± 9.2	69.4 ± 8.3	<0.001
Sex, females	1,235 (53.4%)	1,179 (50.7%)	1,111 (48.3%)	1,127 (50.5%)	0.007
Systolic BP, mmHg	127.7 ± 16.6	127.6 ± 15.7	128.1 ± 16.2	129.8 ± 17.3	<0.001
Diastolic BP, mmHg	77.8 ± 10.5	77.8 ± 10.1	78.0 ± 10.1	78.2 ± 10.7	0.491
FG, mg/dL	96.5 ± 15.1	97.7 ± 16.2	101.3 ± 20.3	119.8 ± 44.7	<0.001
TC, mg/dL	193.9 ± 37.1	195.6 ± 53.7	196.1 ± 39.7	192.7 ± 42.0	0.012
BMI	23.8 ± 2.9	23.9 ± 3.0	23.8 ± 3.0	23.9 ± 3.2	0.673
Hypertension, n (%)	961 (41.5%)	1,000 (43.0%)	1,019 (44.3%)	1,093 (47.1%)	0.001
Diabetes, n (%)	143 (6.2%)	191 (8.2%)	261 (11.3%)	887 (38.2%)	<0.001
Dyslipidemia, n (%)	733 (31.7%)	708 (30.4%)	743 (32.3%)	909 (39.2%)	<0.001
Smoking, n (%)					<0.001
Ex-smoker	268 (12.1%)	306 (13.8%)	335 (15.2%)	279 (12.5%)	
Current smoker	185 (8.4%)	165 (7.5%)	219 (9.9%)	229 (10.3%)	
Heavy alcohol drinker, n (%)	201 (9.1%)	223 (10.1%)	218 (9.9%)	228 (10.2%)	0.021
Regular exercise, n (%)	252 (27.6%)	209 (23.9%)	213 (25.6%)	187 (21.3%)	0.015

Values are presented as mean ± standard deviation or *n* (%).

CV, coefficient of variation; BP, blood pressure; FG, fasting glucose; TC, total cholesterol; BMI, body mass index.

### Incidence of PDD according to glucose variability

During the median 9.5-year follow-up period, 1,757 of 9,264 (19.0%) patients developed PDD. The incidence of PDD increased with increasing glucose variability, from 18.28 per 1,000 person-years in quartile 1 to 20.80 in quartile 2, 22.91 in quartile 3, and 29.14 in quartile 4 (Table 2). In Model 1, higher glucose variability was associated with a higher risk of PDD development after controlling for age, sex, presence of hypertension, diabetes, and hyperlipidemia, heavy alcohol consumption, smoking, and regular exercise. Patients with PD in quartiles 2 (subdistribution hazard ratio [SHR] = 1.30, 95% CI 1.03 to 1.63), 3 (SHR = 1.30, 95% Cl 1.03 to 1.63), and 4 (SHR = 1.50, 95% CI 1.20 to 1.87) had a higher risk of PDD than those in quartile 1 (Table 2). In Model 2, patients with PD in quartiles 2 (SHR = 1.30, 95% CI 1.04 to 1.63), 3 (SHR = 1.29, 95% CI 1.02 to 1.62), and 4 (SHR = 1.50, 95% CI 1.19 to 1.88) independently remained at a higher risk of PDD than those in quartile 1 after additionally controlling for systolic BP, fasting glucose, total cholesterol, and BMI at the index health examination (Table 2).

**Table 2:** Incidence of PDD according to quartiles of glucose variability (CV).

Glucose variability	Patients (*N*)	Event (*n*)	Person-years (PYs)	Incidence rate (per 1,000 PYs)	Model 1^*^	Model 2^**^
quartile 1	2,314	372	20,352	18.28	1 (ref)	1 (ref)
quartile 2	2,327	420	20,190	20.80	1.30 (1.03–1.63)	1.30 (1.04–1.63)
quartile 3	2,301	441	19,292	22.91	1.30 (1.03–1.63)	1.29 (1.02–1.62)
quartile 4	2,322	523	17,949	29.14	1.50 (1.20–1.87)	1.50 (1.19–1.88)

*Model 1 was adjusted for age, sex, presence of hypertension, diabetes and hyperlipidemia, heavy alcohol consumption, smoking, and regular exercise in the Fine and Gray competing regression model including glucose variability as a separate predictor, and death as a competing risk.

**Model 2 was adjusted for all covariates in model 1 plus systolic BP, diastolic BP, fasting glucose, total cholesterol, and BMI at index health examination in the Fine and Gray competing regression model.

PDD, Parkinson’s disease dementia; CV, coefficient of variation; PY, person-year; BP, blood pressure; BMI, body mass index.

### Sensitivity analyses

Among the PD patients without diabetes, those in quartile 4 (SHR = 1.47, 95% CI 1.19 to 1.82) were at a higher risk of developing PDD than those in quartile 1 after controlling for age, sex, presence of hypertension and hyperlipidemia, heavy alcohol consumption, smoking, and regular exercise in Model 1 (Table 3). In Model 2, patients in quartile 4 (SHR = 1.46, 95% CI 1.17 to 1.8) remained independently at a higher risk of PDD than those in quartile 1 after additionally controlling for systolic BP, fasting glucose, total cholesterol, and BMI at index health examination (Table 3).

**Table 3:** SHR for PDD in the patients without diabetes according to glucose variability (CV).

	Model 1^*^	Model 2^**^		
	SHR (95% CI)	*p* value	SHR (95% CI)	*p* value
quartile 1	1 (ref)		1 (ref)	
quartile 2	1.18 (0.96–1.45)	0.120	1.18 (0.96–1.45)	0.120
quartile 3	1.21 (0.98–1.49)	0.084	1.20 (0.97–1.49)	0.088
quartile 4	1.47 (1.19–1.82)	<0.001	1.46 (1.17–1.83)	0.001

*Model 1 was adjusted for age, sex, presence of hypertension and hyperlipidemia, heavy alcohol consumption, smoking, and regular exercise in the Fine and Gray competing regression model, including glucose variability as a separate predictor and death as a competing risk.

**Model 2 was adjusted for all covariates in model 1 plus systolic BP, diastolic BP, fasting glucose, total cholesterol, and BMI at index health examination in the Fine and Gray competing regression model.

SHR, subdistributional hazard ratio; PDD, Parkinson’s disease dementia; CV, coefficient of variation; BP, blood pressure; BMI, body mass index.

In terms of other measures of variability (VIM and ARV), the results showed similar trends. Specifically, using VIM, patients with PD in quartile 4 (SHR = 1.49, 95% CI 1.18 to 1.87) had a higher risk of PDD than those in quartile 1 after controlling for age, sex, presence of hypertension, diabetes and hyperlipidemia, heavy alcohol consumption, smoking, regular exercise, and the index health examination measurements, including systolic BP, baseline fasting glucose, total cholesterol, and BMI (Table 4). For ARV, PD patients in quartiles 3 (SHR = 1.28, 95% CI 1.02 to 1.61) and 4 (SHR = 1.39, 95% CI 1.10 to 1.77) had a higher risk of PDD than those in quartile 1 after controlling for the potential confounders mentioned above (Table 4).

**Table 4:** Incidence rate of PDD according to quartiles of glucose variability (other measures).

Glucose variability	Patients (N)	Event (n)	Person-years (PYs)	Incidence rate (per 1,000 PYs)	Model 1^*^	Model 2^**^
VIM						
quartile 1	2,316	371	20,418	18	1 (ref)	1 (ref)
quartile 2	2,316	410	20,196	20	1.21 (0.96–1.52)	1.21 (0.96–1.52)
quartile 3	2,316	446	19,305	23	1.24 (0.98–1.55)	1.23 (0.98–1.55)
quartile 4	2,316	530	17,864	30	1.48 (1.19–1.85)	1.49 (1.18–1.87)
ARV						
quartile 1	2,195	342	19,301	18	1 (ref)	1 (ref)
quartile 2	2,423	429	21,098	20	1.12 (0.89–1.42)	1.11 (0.88–1.41)
quartile 3	2,309	460	19,414	24	1.29 (1.02–1.62)	1.28 (1.02–1.61)
quartile 4	2,337	526	17,971	29	1.40 (1.11–1.76)	1.39 (1.10–1.77)

*Model 1 was adjusted for age, sex, presence of hypertension, diabetes and hyperlipidemia, heavy alcohol consumption, smoking, and regular exercise in the Fine and Gray competing regression model, including glucose variability as a separate predictor and death as a competing risk.

**Model 2 was adjusted for all covariates in model 1 plus systolic BP, diastolic BP, fasting glucose, total cholesterol, and BMI at index health examination in the Fine and Gray competing regression model.

PDD, Parkinson’s disease dementia; PY, person-year; VIM, variability independent of mean; ARV, average real variability.

## Discussion

This large-scale nationwide cohort study investigated the association between glucose variability and PDD incidence. Our results found that a higher visit-to-visit glucose variability was associated with a greater incidence of PDD in patients with PD, regardless of baseline fasting glucose levels. In particular, the effects of higher glucose variability remained valid in patients without diabetes. These findings present higher glucose variability as a modifiable risk factor for incident PDD. Therefore, prevention strategies that include controlling glucose levels are needed to protect against PDD development in patients with PD.

Recent studies on the identification of prognostic factors related to various complications in patients with diabetes have focused on glucose variability, as well as continuous hyperglycemia or hypoglycemic events, and found it to be associated with cognitive impairment (Takao et al., 2014), Alzheimer’s disease (Li et al., 2017; Zheng et al., 2021; Lee et al., 2022), and vascular dementia (Lee et al., 2022). Additionally, among individuals without diabetes, glucose variability has been reported to increase the risk of both cognitive impairment (Bancks et al., 2018) and Alzheimer’s disease (Lee et al., 2018). However, there is a paucity of information on the effects of glucose variability on poor clinical outcomes, including the development of dementia in patients with PD.

Our novel finding was that long-term glucose variability, a reflection of residual insulin secretion and the stability of medication effect and adherence, is a risk factor for PDD, regardless of other cardiovascular risk factors and baseline glucose levels. In particular, the increased risk of PDD remained in the highest quartile of glucose variability among patients without diabetes, indicating that glucose variability might affect the development of PDD independently of continuous hyperglycemia and hypoglycemic events. Furthermore, patients in the highest quartile of glucose variability consistently had a higher risk of PDD than those in the lowest quartile using all three variability indices (CV, ARV, and VIM). While further studies are needed to elucidate these findings, several mechanisms might explain the impact of glucose variability on the development of dementia in patients with PD. Previous studies have shown that, compared with consistent hyperglycemia, glucose variability exerts more deleterious effects on inflammatory reactions, oxidative stress, and endothelial dysfunction, which are closely related to neurodegeneration (Quagliaro et al., 2005; Ceriello et al., 2008). Glucose variability has also been found to cause insulin resistance and cerebral small vessel damage, thus hindering the clearance of pathogenic proteins in the brain (Keating and El-Osta, 2013; Takao et al., 2014). Shared mitochondrial dysfunction may offer an alternative explanation. Striatal neurons and pancreatic β-cells have low mitochondrial capacity and are vulnerable to dysfunctions in mitochondrial respiratory chain enzyme (Schernhammer et al., 2011). Furthermore, growing evidence has shown that insulin signaling pathway disruptions may play a major role in the pathogenesis of PD, including inflammation, oxidative stress, and increased α-synuclein deposition in the brain, which are associated with poor prognosis (Kim et al., 2011; Athauda and Foltynie, 2016). In fact, insulin receptor mRNA expression in the striatum was lower in the brain tissues of patients with PD than in those without PD.

The strengths of this study lie in the large number of patients with PD and the long follow-up duration in a nationwide cohort. However, this study also had several limitations that must be addressed. First, discrepancies in the diagnosis of PD and PDD between clinical practice and the KNHIS claim data may have produced inaccurate results. PDD was defined according to ICD-10 codes (F00, F02, F03, F05 or G30) in PD patients, although F00 and G30 refers to dementia of the Alzheimer type. As the KNHIS covers the dementia medication (donepezil, rivastigmine, galantamine, and/or memantine) for only patients with dementia of the Alzheimer type, most Korean doctors put F00 or G30 codes for patients with PDD to prescribe the dementia medication covered under health insurance. However, this issue might be mitigated by the inclusion of the diagnostic code of PD in the registration code in the program for rare intractable diseases to increase diagnostic accuracy. Second, due to the limitation of claims data, we were unable to assess the potential confounders including glycosylated hemoglobin, exact cognitive performance at baseline, neuroimaging finding, concomitant cerebrovascular disease, education years, PD motor subtype, and compliance with PD medication of each patient. This was partially overcome by the exclusion of subjects with a prior diagnosis of dementia and diagnosis of dementia within the first year of follow-up. Third, although fasting glucose level may be affected by the meal consumed the night before blood sampling, we could not evaluation this information. Finally, we did not account for the effects of glucose-lowering medications and compliance with glucose-lowering medications. However, we defined diabetes according to the diagnostic code and prescriptions of glucose-lowering medication and adjusted our analyses for the presence of diabetes, which may have mitigated this issue to a certain extent. Despite these limitations, our study successfully identified the effect of long-term glucose variability on cognitive decline in patients with PD. Our findings suggest that preventive measures for constant glucose control may be necessary to prevent PDD.

## Data availability statement

Publicly available datasets were analyzed in this study. This data can be found at NCBI, accession numbers: ERP110230 and PRJNA517480.

## Ethics statement

This study was approved by the Institutional Review Board of the Korea University Guro Hospital and adhered to the principles of the Declaration of Helsinki. The studies were conducted in accordance with the local legislation and institutional requirements. The ethics committee/institutional review board waived the requirement of written informed consent for participation from the participants or the participants' legal guardians/next of kin because Anonymous and de-identified information was used for analysis; therefore, informed consent was not obtained.

## Author contributions

SK: Conceptualization, Data curation, Investigation, Methodology, Writing – original draft, Writing – review & editing, Funding acquisition, Validation. YC: Data curation, Formal analysis, Writing – review & editing. SC: Data curation, Writing – review & editing. S-JM: Formal analysis, Writing – review & editing. CK: Data curation, Writing – review & editing. JK: Data curation, Writing – review & editing. KO: Data curation, Writing – review & editing. JY: Data curation, Writing – review & editing. SS: Data curation, Writing – review & editing. GC: Data curation, Writing – review & editing. S-BK: Data curation, Methodology, Project administration, Supervision, Writing – review & editing.

## Footnotes

^1^ http://nhiss.nhis.or.kr
